# Potential Roles of *Stevia rebaudiana* Bertoni in Abrogating Insulin Resistance and Diabetes: A Review

**DOI:** 10.1155/2013/718049

**Published:** 2013-11-12

**Authors:** Nabilatul Hani Mohd-Radzman, W. I. W. Ismail, Zainah Adam, Siti Safura Jaapar, Aishah Adam

**Affiliations:** ^1^Faculty of Pharmacy, Universiti Teknologi MARA, Puncak Alam Campus, 42300 Bandar Puncak Alam, Selangor Darul Ehsan, Malaysia; ^2^Medical Technology Division, Malaysian Nuclear Agency, Bangi, 43000 Kajang, Malaysia

## Abstract

Insulin resistance is a key factor in metabolic disorders like hyperglycemia and hyperinsulinemia, which are promoted by obesity and may later lead to Type II diabetes mellitus. In recent years, researchers have identified links between insulin resistance and many noncommunicable illnesses other than diabetes. Hence, studying insulin resistance is of particular importance in unravelling the pathways employed by such diseases. In this review, mechanisms involving free fatty acids, adipocytokines such as TNF**α** and PPAR**γ** and serine kinases like JNK and IKK**β**, asserted to be responsible in the development of insulin resistance, will be discussed. Suggested mechanisms for actions in normal and disrupted states were also visualised in several manually constructed diagrams to capture an overall view of the insulin-signalling pathway and its related components. The underlying constituents of medicinal significance found in the *Stevia rebaudiana* Bertoni plant (among other plants that potentiate antihyperglycemic activities) were explored in further depth. Understanding these factors and their mechanisms may be essential for comprehending the progression of insulin resistance towards the development of diabetes mellitus.

## 1. Introduction

The emergence of many non-communicable diseases has been prominent over the last century. One of the global epidemics is diabetes, that has progressively affected the human populations for 20 centuries.

Diabetes mellitus is a metabolic disorder signified by high levels of glucose in the blood and can be categorised into two main groups. The first group (Type I) is often used to describe the onset of diabetes, which is triggered by the inability of the pancreas to produce sufficient amounts of insulin for glucose uptake and metabolism. Leney and Tavaré [1] report that the insufficiency of insulin in Type I diabetes results from the destruction of the autoimmune response, which disrupts the pancreatic *β*-cells.

The second group is noninsulin-dependent diabetes mellitus (NIDDM), also referred to as Type II diabetes, which is primarily related to insulin resistance. Many researchers agree that Type II diabetes is predominantly caused by impairment of the insulin-signalling pathway, even though the exact disease pathogenesis is yet to be understood. Even so, insulin resistance has been closely related to reduced metabolic responsiveness to normal insulin circulation [2]. Additionally, insulin resistance involves an abnormal biological response of the body systems with regard to physiological levels of insulin, and this pathological feature of the disease is the key to the metabolic syndrome.

There have also been reported cases in the American population of increased susceptibility to Type II diabetes due to family history and lack of cardiorespiratory fitness [3]. Although Type II diabetes has been asserted to have a genetic linkage [4], the key here is insulin resistance, which can be exacerbated by lifestyle changes and unhealthy dietary intake [3]. DeFronzo [5] states that insulin resistance and Type II diabetes have been linked to clusters of cardiovascular and metabolic disorders including hypertension, obesity, glucose intolerance, dyslipidemia, and endothelial dysfunction. Apart from that, Type II diabetes has also been referred to as obesity-associated insulin resistance and implicated in the development of hypertension and atherosclerosis.

Theoretically, insulin resistance is defined as a state in a cell, tissue, system, or body for which levels of insulin needed to produce a quantitatively normal response are greater than normal. It is claimed that insulin has diverse effects and actions, depending on the different types of cells and tissues it is reacting to in the body. Insulin resistance is also closely related to hyperinsulinemia, though high blood glucose is observed in the former while high insulin is observed in the latter. Insulin resistance also occurs in clinical settings such as pregnancy, cancer cachexia, obesity, starvation, burn trauma, sepsis, and as an outcome of several experimental treatments, both *in vivo* and *in vitro* [6]. Insulin-mediated glucose disposal is essentially impaired in most identified cases, as glucose levels are the main feedback signal for compensatory hyperinsulinemia [7, 8]. The past 20 years have seen various schemes put forward to categorise the different mechanisms of insulin resistance with respect to the diverse molecular pathways involved.

Aside from that, the prevalence of metabolic syndromes like insulin resistance has triggered a quest for developing alternative treatments, if not new drugs, for these diseases. Communities around the world, particularly in rural areas, have been practising folk medicines using their own local resources. In the case of diabetes, many herbs and fruits with antihyperglycemic effects have been studied, prompted by their use in folk medicines, particularly those from the tropical and Asian regions. For example, in Bangladesh, leaves from several species of fruit tree have been tested for their ability to reduce serum glucose levels in mice; these include *Averrhoa carambola*, *Ficus hispida*, and *Syzygium samarangense* [9]. Fruit peels have also proved to have anti-hyperglycemic properties, as shown by the evaluation of blood glucose levels in Wistar rats fed with raw *Psidium guajava* (guava) fruit peels [10]. *Cynodon dactylon* is another example; a weed, known and popularised by the name “Doob” in India, has been found to be highly potent in its anti-hyperglycemic activities, as observed in Streptozotocin-induced diabetic rats [11].

Indirectly, these studies support the development of natural products from plant extracts and fruit products as sources of hypoglycemic agents and potential alternatives to off-the-shelf antidiabetic drugs. Among the many herbs prevalent in ancient and traditional folk medicine practices, *Stevia rebaudiana* Bertoni, a perennial shrub from South America, is a prime example [12]. The sweetness and unique properties of this plant provide an interesting platform for revealing its potential medicinal effects, specifically with regard to insulin resistance.

## 2. Insulin-Signalling Pathway

In order to tackle insulin resistance, it is important to understand the major insulin-signalling pathways involved and their impact on the regulation of blood glucose levels. Since the discovery of insulin, its correlation with fat, carbohydrate, and protein metabolism has been well established, but the molecular mechanisms of its actions remain difficult to define. It is well understood that insulin action begins with the binding of insulin to its receptor. The insulin receptor (IR) protein has been described as a heterotetramer of two identical extracellular *α*-subunits and two transmembrane *β*-sub-units which span the cell membrane [13]. The *α*-chain lies in the extracellular portion of the cell membrane, while the *β*-chain spans the cell membrane in a single transmembrane segment, with parts of it lying in the intracellular compartment of the membrane, and these are all held together by disulphide bonds [14]. This insulin receptor is a tyrosine kinase that has two different ligand binding regions: a high-affinity site and a low-affinity site [15].

Physiologically, insulin triggers the insulin receptor into exerting its effects, leading to the phosphorylation of insulin receptor substrate (IRS) proteins [16]. On detection of high levels of glucose released from the ingestion and uptake of food into the body, *β*-cells in the pancreas will first release insulin in response, which is prior to the binding of insulin to specific cell surface receptors. These specific cell receptors are embedded in the cell membrane of a fat, brain, or muscle cell. The binding will then lead to the activation of “second messengers” acting as intracellular mediators, initiating and stimulating a cascade of phosphorylation and dephosphorylation activities, which are responsible for a series of pathways and metabolic mechanisms, including glucose transport.

The first step in the cascade involves the activation of the IR tyrosine kinase by the autophosphorylation of the *β*-sub-unit, via the self-addition of phosphate groups in the intracellular domain of the receptor. This causes a conformational change in the receptor, which will also help in other adenosine triphosphate (ATP) binding and facilitate the gathering of other substrates for the ensuing phosphorylation activities. Phosphorylation of insulin receptor substrate 1 (IRS1) together with other intracellular substrates will ensue later, via the action of the activated IR tyrosine kinase.

Generally, these phosphorylated substrates will each provide unique docking sites for particular effector proteins with Src homology 2 (SH2) domains, which will recognise those residues with high specificity—and in this case, the SH2 domain of the phosphoinositide 3-kinase (PI3-K) will specifically identify the phosphotyrosine residue of IRS1 and will subsequently bind with it, passing down the signal for the next step in the signalling pathway.

**Figure 1:**
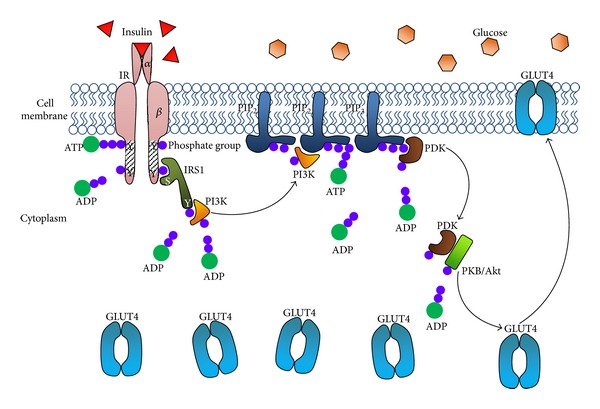
Manually derived and structured mechanisms of the insulin-signalling pathway in a normal state triggered by high glucose levels in the blood, prompting insulin binding and cascades of phosphorylation by ATP bindings, finally leading to the migration of GLUT4 from the cytoplasm to the cell membrane for extracellular glucose uptake. IR: insulin receptor; Y: tyrosine; S: serine; ATP: adenosine triphosphate; ADP: adenosine diphosphate; IRS1: insulin receptor substrate 1; PI3K: phosphoinositide kinase 3; PIP_2_: phosphatidylinositol 4,5-bisphosphate; PIP_3_: phosphatidylinositol 3,4,5-trisphosphate; PDK: PIP_3_-dependent kinase; PKB/Akt: protein kinase B; GLUT4: glucose transporter 4.

Kinases are important components of signalling pathways and phosphorylation, in terms of transmitting the signal from one compartment to the other. In this mechanism, the signal corresponds to the level of blood glucose and is transmitted from the extracellular environment to the intracellular cavity. Following the binding process, the enzyme is activated, thus triggering the PI3-K pathway later leading to the phosphorylation of phosphatidylinositol-(4,5)-bisphosphate (PIP_2_) into phosphatidylinositol-(3,4,5)-trisphosphate (PIP_3_). The generation of PIP_3_ activates sets of specific proteins, enzymes, substrates, and molecules; and this includes phosphoinositide-dependent kinase 1, which initiates a number of downstream proteins including protein kinase B (PKB) or Akt. The activation of Akt through its translocation to the membrane is directly assisted by PIP_3_ via the pleckstrin homology domain. Akt has an important and central role in insulin-stimulated glucose uptake, as it is a major target in PI3-K activities, directly associating upstream insulin signalling with Glucose Transporter 4 (GLUT4) translocation [17]. Metabolic enzymes like glycogen synthase kinase 3 and 6-phosphofructo-2-kinase are regulated by Akt activation—apart from it stimulating the translocation of GLUT4 to the plasma membrane from the intracellular storage compartment, in order to take up the ingested extracellular glucose (Figure 1) [17].

## 3. Molecular Mechanisms of Insulin Resistance

The involvement of insulin resistance in diabetes was initially proposed in 1939 by Sir Harold Percival Himsworth, a British scientist [18]. Previously, diabetes was believed to be caused only by the deficiency of insulin. Since this breakthrough, the research on insulin resistance (particularly its molecular mechanism) is still progressing, mainly with regard to fatty acids, adipocytokines like tumour necrosis factor  (TNF*α*), peroxisome proliferator activator receptor *γ* (PPAR*γ*), and serine kinases like c-Jun NH_2_-terminal kinase (JNK) and the inhibitor of nuclear factor *κ*B kinase *β* (IKK*β*).

### 3.1. Free Fatty Acids

The mediation of insulin resistance in tissue remains complex and difficult to define, but researchers have found that it can be facilitated by factors such as free fatty acids (FFAs). Fat accumulation has been strongly linked to elevated glucose production and insulin resistance and hence to increased susceptibility to Type II diabetes [19]. Based on the observations of Savage et al. [2], there is a strong correlation between circulating FFAs and obesity and insulin resistance, which supports this hypothesis. In addition, the elevation of FFA levels observed *in vitro* among 3T3-L1 adipocytes caused mitochondrial dysfunction in the cells, apart from causing decreased insulin-stimulated glucose uptake [20]. The same occurrences were observed *in vivo* in Zucker *fa/fa* rats, where FFA levels were proportional to the levels of reduced insulin-mediated glucose uptake [21]. Adipose cells and tissues are of particular importance in this case, as they administrate fat confiscation in whole-body metabolism processes, which also links high concentrations of FFAs and triglycerides circulation with the deficiency in adipose tissues [22].

**Figure 2:**
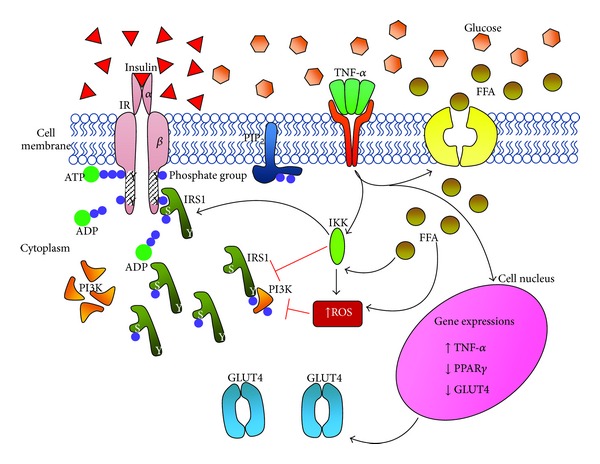
The disruptions in the insulin-signalling pathway in an insulin-resistant state caused by elevated actions of TNF-*α* and FFA. IRS1 is no longer phosphorylated on its tyrosine residues but on serine residues, resulting in nonfunctional, inhibitory proteins. TNF-*α* also influences increased gene expressions of TNF-*α* but decreases PPAR*γ* and GLUT4 expressions, resulting in lower levels of GLUT4 proteins. Glucose uptake is reduced, leading to hyperglycemia and hyperinsulinemia. IR: insulin receptor; Y: tyrosine; S: serine; ATP: adenosine triphosphate; ADP: adenosine diphosphate; IRS1: insulin receptor substrate 1; PI3K: phosphoinositide kinase 3; PIP_2_: phosphatidylinositol 4,5-bisphosphate; TNF-*α*: tumour necrosis factor *α*; FFA: free fatty acid; IKK: a type of serine kinase; ROS: reactive oxygen species; PPAR*γ*: peroxisome proliferator activator-receptor *γ*; GLUT4: glucose transporter 4.

Moreover, FFAs were also revealed to have induced insulin resistance by initially disrupting the phosphorylation process in the insulin-signalling pathway and consequently reducing glucose oxidation and glycogen synthesis (Figure 2) [23]. Reduced glucose oxidation and glycogen synthesis increase FFA oxidation, which causes an increase in and accumulation of glucose-6-phosphate, inhibiting the action of hexokinase II in the glycogen synthesis pathway [23]. Such inhibitory effects cause the glucose level in the cells to increase, prompting glucose uptake to halt; thus, the glucose levels in the bloodstream will also rise. This will eventually lead to insulin resistance and diabetes, as a long-term impact.

Similar observations have been recorded where increased FFA oxidation has led to increased reactive oxygen species (ROS) levels, which may lead to increased fat accumulation [24]. This strongly supports the theory that a pro-oxidant environment corresponding to metabolic disorders like insulin resistance may have been hugely influenced by the inability of the cells to combat oxidative stress. Furthermore, in a study implementing the TNF*α* cytokine, it was suggested that JNK might be the mediator to ROS-induced insulin resistance [6]. Both JNK and TNF*α* play major roles in the progression of insulin resistance and are discussed later in this review.

These observations also suggest that ideal glucose homeostasis and insulin sensitivity go hand in hand with sufficient adipose tissue with respect to a person's body size. The importance of adipose tissue in a body's metabolism activities and in homeostasis is emphasised by the fact that adipose tissues play a major role in adipokines secretion.

### 3.2. Tumour Necrosis Factor *α* (TNF*α*)

The functionality of an adipocyte's role as an endocrine cell to secrete biologically active enzymes and proteins such as adipokines increases as a result of an elevation in adiposity. An example of these adipokines is TNF*α*, which has a major role in inflammatory responses in the cell. TNF*α* is recognised as a multifunctional proinflammatory cytokine, which is expressed as a 26-kDa transmembrane prohormone and produces a 17-kDa soluble form of the TNF*α* molecule on proteolytic cleavage. It also performs countless biological functions in the cell. Recently, TNF*α* has become the main focus of this particular field of research, the aim being to unravel its physiological and pathophysiological functions, which include transcriptional regulation, fatty-acid metabolism, hormone-receptor signalling, glucose metabolism, and adipocyte differentiation. Many current studies have concluded that TNF*α* actions and contributions to the system are implicated in metabolic disturbances like obesity and insulin resistance. This provides a platform for the implementation and use of TNF*α* in insulin-resistance studies, as a factor to induce insulin resistance in the cells of interest.

TNF*α* is often found in adipose tissue as well as in human fat, and the levels of its mRNA have been closely linked to the prevalence of hyperinsulinemia and obesity. In *in vivo* testing, a decrease in TNF*α* level is correlated to a loss in body weight [25]. The basis of this hypothesis is that TNF*α* was found to contribute to the induction of insulin resistance, although its exact mechanism of action is yet to be established. As a proinflammatory cytokine, TNF*α* is responsible for the development of metabolic syndromes and the maintenance of metabolic homeostasis, exerting its actions through the immune and inflammatory pathways [26]. States indicative of Type II diabetes were indicated by insulin resistance induced by TNF*α*, through the inhibition of tyrosine (tyr) phosphorylation of IRS1 [27] (Figure 2). Sethi and Hotamisligil [28] report that TNF*α* is highly responsible in lipid metabolism, where its increased levels are directly proportional to the increased levels of basal lipolysis, a major biochemical site in this process (Figure 2). In both *in vitro* and *in vivo* states, lipolysis can be initiated—together with an elevation of circulating free-fatty-acid concentrations—through the administration of exogenous TNF*α*. In addition to that, TNF*α* has the ability to inhibit lipoprotein lipase (LPL) activities that occur in fatty-acid uptake derived from lipolysis [29].

Expressions of free-fatty-acid transporters were also claimed to be reduced, resulting in reduced FFA uptake, which leads to hyperlipidemia—all in the course of TNF*α*'s actions. This implies that TNF*α* also controls the escalations in lipolysis that lead to hyperlipidemia. There were also reduced expressions of key enzymes like acetyl-CoA carboxylase, fatty-acid synthase, and acyl-CoA synthetase, all of which affect insulin-mediated glucose uptake. Moller [21] mentions similar states, in which TNF*α* suppressed the expression of gene-encoding proteins with the likes of acetyl-CoA carboxylase and LPL, in charge of lipogenesis. Moller [21] also suggests that downregulation of GLUT4 (among other metabolic components) may lead to better understanding of the mediation of TNF*α* in inducing insulin resistance.

Previously, it has been theorised that TNF*α* is responsible for a number of catabolic states, such as sepsis, burn trauma, and cancer. TNF*α* produces its effects through several actions targeting insulin sensitivity: insulin receptor signalling, glucose transport, improved lipid metabolism, and leptin production [28]. The main mechanisms of TNF*α* actions are yet to be defined, but it appears to be able to downregulate GLUT4 directly (based on significantly reduced GLUT4 mRNA levels, documented after TNF*α* treatment) [30]. Phosphorylation of IRS1 at serine residues instead of tyrosine (which blunts insulin signalling) was observed to have increased via the induction of TNF*α* in cultured adipocytes. These incidences caused a conformational change in the multifunctional docking protein, forcing it to inhibit the insulin receptor (IR) tyrosine kinase on its binding site. The correlation between IRS1 and PI3-K in the downstream events of insulin signalling is also reduced due to this IRS1 modification. Further evidence includes an increase in insulin-signalling activity and efficiency by the effective genetic and pharmacological blockade of TNF*α* actions, observed *in vivo* on rat specimens [31].

### 3.3. Peroxisome Proliferator-Activator Receptor *γ* (PPAR*γ*)

Another factor worthy of mention here would be PPAR*γ*, which serves as an important function in adipocyte functionality and is a major transcriptional regulator in adipogenesis. PPAR*γ* works together with the CCAAT/enhancer-binding protein (C/EBP) family of transcription factors in the regulation of adipogenesis. PPAR*γ* can also react with insulin sensitisers, which serve as its agonists and ligands [32]. Therefore, many researchers have been targeting PPAR*γ* pharmacologically for drug developments, especially concerning diabetes. PPAR*γ* agonists have been studied in the past; they were given as treatments both *in vitro* and *in vivo*, resulting in normalised serum insulin and glucose concentrations in insulin-resistance models [33]. PPAR*γ* showed great potential for sensitising the insulin-signalling pathway in the observed insulin-resistant states.

Apart from its insulin-sensitising activities, PPAR*γ* also plays a role in adipocyte differentiation. There have been numerous studies demonstrating relationships between the posttranscriptional covalent modifications of PPAR*γ* (through phosphorylation and sumoylation of said protein) to the progression of metabolic deteriorations, including diabetes. Researchers have established that one of the ways to combat insulin resistance and diabetes therapeutically is to tackle the covalent modifications of PPAR*γ*, though more extensive studies need to be done [34]. Nonetheless, pharmacological functions of PPAR*γ* in promoting glucose uptake were seen to have been restored, with the prevention of sumoylation in 3T3-L1 adipocytes [35].

Another potential line of enquiry is that PPAR*γ* is closely linked to TNF*α*, as TNF*α* can downregulate the expression of PPAR*γ* in observed 3T3-L1 adipocytes [36]. The fact that PPAR*γ* facilitates the maintenance of normal insulin sensitivity leads to the conclusion that its inhibition by TNF*α* could possibly account for TNF*α*-induced insulin resistance. In addition, this finding is backed up by a study showing PPAR*γ* agonists averting lipolysis and preventing an increased elevation of FFAs in 3T3-L1 cells that were initially subjected to the actions of TNF*α* [37]. PPAR*γ* transcriptional activities are also affected by treatments with antidiabetic drugs such as thiazolidinediones and pioglitazones [38], and all of these indirectly support its involvement and importance in maintaining normal glucose homeostasis.

### 3.4. Serine Kinases

Several studies on TNF*α* plausibly connect TNF*α* with influencing ROS levels in cells [6]. Both ROS and TNF*α* are reported to be able to cause insulin resistance and to be potent activators of JNK—also conceivably a factor contributing to the metabolic syndrome. Guo et al. [16] explain that the inhibitory effects of both ROS and TNF*α* on insulin sensitivity might involve several serine/threonine kinase cascades, which suggest the implication of JNK and IKK*β* as major candidates. The significance of and possibilities for JNK mediating the role of TNF*α* in insulin resistance were also proposed in this study. Guo et al. [16] report that levels of JNK activation and the phosphorylation of IRS1 on its serine residues were significantly increased with *in vitro* TNF*α* treatment in 3T3-L1 cells. The authors elaborate on the role of JNK; they observed a prevention of insulin resistance with the gene knock-down of JNK1 protein expression. Apart from that, insulin sensitivity of the 3T3-L1 adipocytes treated with TNF*α* was also seen to have improved, merely by inhibiting JNK activation.

Houstis et al. [6] provide a similar take on this; in their studies, decreased phospho-JNK levels in the 3T3-L1 adipocytes led to an elevation of insulin-mediated glucose uptake. JNK normally functions as a sensing juncture for inflammatory status and cellular stress, but it also targets the serine (ser-307) site of IRS1, as it also vigorously phosphorylates IRS1 on that particular site [27]. As phosphorylation on this site produces blunt insulin signalling, the presence and action of JNK in facilitating this process further decrease insulin sensitivity and lead to insulin resistance.

**Figure 3:**
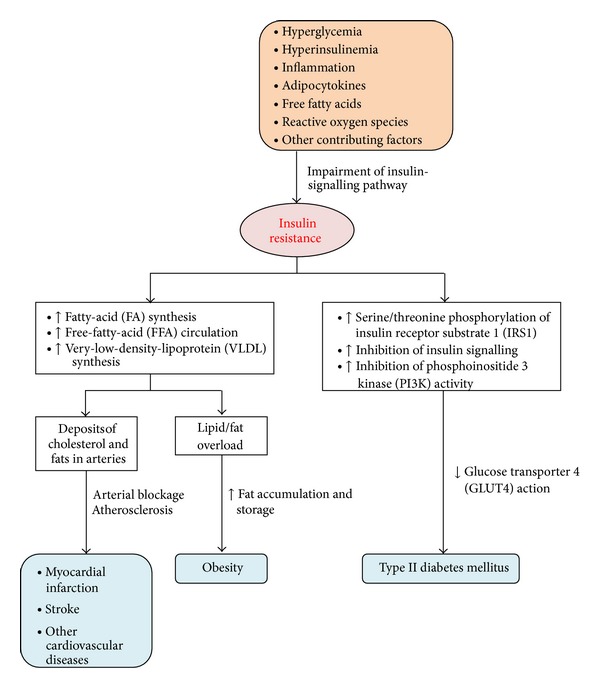
Manually constructed flowchart summarising the factors leading to insulin resistance that will eventually result in many related diseases.

Because of current findings linking insulin resistance to diabetes, many researchers are now focusing on the diverse molecular mechanisms in the progressions of both metabolic conditions, which underlines the importance of understanding the metabolic syndrome. Additionally, adipocytokines like TNF-*α*, serine kinases and free fatty acids are among the many factors and channels that may contribute to insulin resistance, type II diabetes mellitus, and other diseases (Figure 3 and Table 1).

## 4. Stevia as an Antidiabetic Agent

To date, out of the 150 known species of Stevia, *Stevia rebaudiana* Bertoni is the only one of its kind found to have particular attributes: firstly, it is unique in the potency of its sweetness [39]. Furthermore, this particular plant has been used by the Guarani Indians of Paraguay and Brazil to treat diabetes, due to its therapeutic qualities [12]. Even though the plant's leaves give out a distinctly sweet taste, they contain no calories [40], though they are rich in metabolites such as *β*-carotene, thiamine, austroinulin, riboflavin, diverse terpenes, and flavonoids, which give the plant its medicinal advantages [41]. This zero-calorie property can also be beneficial to patients suffering from obesity and diabetes, as it will not elevate their blood-glucose levels. Contrast this with the effects of sucrose (normally extracted from sugar beets and sugar cane), which may cause stomach infections and dental caries [39].

On the whole, researchers worldwide agree on the antidiabetic effects of Stevia; but they differ on *how* the effects contribute towards combating this metabolic disease. It is important to note that there are many steviol glycosides, which are compounds with multiple carbohydrate molecules, bound to a noncarbohydrate, aglycone moiety (steviol) that can be extracted from the *Stevia rebaudiana* Bertoni plant, most commonly are stevioside, rebaudioside A, rebaudioside C and dulcoside, among many other available glycosides [42, 43]. Some assert that Stevia's utility is due to its antioxidant properties; this is supported by analysis of the phenols that may be extracted from the plant. Stevia has a large overall proportion of phenols, up to 91 mg/g; it is proposed that these constituents extracted from the leaves are the major agents contributing towards the antihyperglycemic activities exerted by the plant [44]. This is further supported by the fact that the leaves have a greater ability to scavenge free radicals and prevent lipid peroxidation than controls such as butylated hydroxytoluene, butylated hydroxyanisole, and tertiary butyl hydroxyquinone [44].

Such findings concur with the results of other studies of Type 1 diabetes, modelled by streptozotocin-induced diabetic rats, in which phenolic compounds prevented several diabetic complications [45]. In addition, Shivanna et al. [44] observed a significant decrease (about 30%) in peroxidation in the livers of Stevia-pre-fed rats, compared to those of their control groups. This is a good indicator of reduction in the progression of diabetic complications, as diabetic tissue damage is commonly linked to the peroxidation of lipids, likewise the condition of hyperglycemia, which increases the production of reactive oxygen species (ROS) in the tissues due to high blood-glucose levels, which makes the tissues susceptible to oxidation [46].

### 4.1. Maintenance of Blood-Glucose Levels

As previously discussed, it is highly likely that Stevia's antioxidants are the source of its most medicinally beneficial effects. One such effect is the maintenance of blood-glucose levels, which is the most common measure used by researchers to evaluate the effectiveness of an anti-hyperglycemic agent. Susuki et al. [47] observed a significant decrease in blood-glucose levels over four weeks in rats fed with Stevia (combined with high-carbohydrate and high-fat diets). Similarly, NMRI-Haan laboratory mice induced to hyperglycemia using glucose experienced a significant reduction in glycemia after a week's treatment with stevioside, a major component of the leaf extract [47]. The same trend was seen in those assigned to the adrenaline load test after the same treatment periods.

It was further reported that there was a significant reduction (an average of 18%) in postprandial glucose levels in Type II diabetic patients given test meals supplemented with stevioside [48]. A study by Anton et al. [49] confirmed this; postprandial glucose levels were significantly lowered in patients supplemented with Stevia, compared to those given aspartame (a type of synthetic sweetener) or sucrose (normal table sugar). Interestingly, patient satiety as an after-effect of the different sweeteners was also tested; it was found that subjects given lower-calorie sweeteners (Stevia or aspartame) did not compensate by eating more than those given sucrose.

### 4.2. Anti-Inflammatory Response

A study on the C57BL6J insulin-resistant mice model shows that stevioside is also able to downregulate the nuclear factor *κ*-light-chain enhancer of activated B cells (NF-*κ*B) pathway, as well as enhancing whole-body insulin sensitivity, glucose infusion rate, and the level of the glucose-lowering effect of insulin [40]. Additionally, and interestingly, the expression of TNF*α* (the previously discussed proinflammatory cytokine contributing to the reduction of insulin sensitivity) was significantly downregulated, together with the expressions of interleukin 6 (IL6), interleukin 1*β* (IL1*β*), and interleukin 10 (IL10), among other chemotactic and pro-inflammatory cytokines [40]. Therefore, stevioside was seen to be able to potentiate in the reduction of insulin resistance through reducing the inflammation in adipose tissues by regulating TNF*α*.

### 4.3. Influence on Insulin Secretion

Various hypotheses have been constructed as to how stevioside causes such significant reductions in blood-glucose levels; these include theories of glucose disposal [50], modulation of glucose transport [51], and improvement in insulin sensitivity and secretion [52]. Jeppesen et al. [53] first reported that insulin release can be directly influenced by both stevioside and steviol solely, as they observed an increase in insulin secretion in both the INS-1 pancreatic *β*-cell line and in normal mouse islets. They also hypothesised that stevioside may only exert its glucose-depleting effects in specific high-blood-glucose conditions (as in a diabetic setting), as they failed to prove otherwise in non-hyperglycemic conditions [48]. This is a very encouraging finding; it signifies that stevioside can be target-specific, lowering glucose levels at specific settings without jeopardising the patient's health by risking severe hypoglycemia.

### 4.4. Insulinotropic, Glucagonostatic, and Nutrient-Sensing Effects

With the regulation of hormones with the likes of insulin comes nutrient sensing, which is literally what the term suggests: an organism's ability to sense and target available nutrients in order to control and regulate the related metabolic pathways and fluxes. Unlike prokaryotes that can manage their own nutrient sensing, eukaryotes are more complex, in terms of the influences of nutrient availability on the metabolic processes (particularly by both neuronal and hormonal signal transductions, such as glucagon and insulin). In recent years, it has been shown that nutrient sensing can operate both autonomously and in coordination with other endocrine pathways as a response to macronutrient fuel substrates such as glucose, amino acids, and lipids [54]. These pathways are essential to the regulation of cellular homeostasis, full utilisation of available nutrients, and for survival during starvation [55].

In a more recent publication by Jeppesen et al. [56], the authors state that stevioside contributes to insulinotropic and glucagonostatic effects by increasing insulin secretions while suppressing glucagon, apart from being anti-hyperglycemic to Goto-Kakizaki (GK) rats, as non-obese Type II diabetic animal models. Insulin depletion and elevation in glucagon levels in a Type II diabetes condition have been closely linked with dysfunction in the *α*-pancreatic cells, contributing (along with the more commonly implicated culprit, insulin resistance) to the development of the disease [57]. This supports the theory of Jeppesen et al. [58] that stevioside's glucagonostatic effects might be brought about by an indirect insulin-induced inhibitory response to glucagon, the increase in effectiveness of glucose recognition, or a straightforward inhibition of glucagon production by the *α*-pancreatic cells.

Furthermore, elevated levels were observed of the genes responsible for glycolysis, which may have contributed to the elevated insulin secretions. This is also thought to improve nutrient sensing in the specimen, as is the downregulation of proteins such as phosphodiesterase 1 (PDE1), responsible for the cyclic adenosine monophosphate (cAMP) degradation concomitant with stevioside treatments. In cases where PDE1 is downregulated, cAMP (essential in amplifying insulin secretions physiologically induced by glucose) will be increased, suggesting stevioside's ability to holistically amplify the expressions of glucose-responsive genes and improve nutrient sensing [58].

## 5. Conclusion

Research in this field has established that the metabolic syndrome encompassing diabetes, obesity, and insulin resistance is highly correlated to various aspects, from the selection of a cell culture model through the understanding of each and every step in the mechanisms involved, with proper comprehension of the function of each component on the pathways. In order to counter the metabolic syndrome as a whole, it is essential to go through all the tiny details of each metabolic process. Even so, it is essential for researchers to look into the potential healing ability (bestowed on us by nature, but often well hidden) of diverse herbs and plants.

It is postulated that the *Stevia rebaudiana* Bertoni plant could benefit the community medicinally through several different pathways, all eventually leading to its anti-hyperglycemic qualities. Although there are many unknowns and anomalies in our knowledge of insulin-signalling pathways, the mechanisms of glucose uptake, and the metabolic processes involved in insulin resistance, these loopholes could be addressed if researchers were to focus more on key factors such as IRS1, its phosphorylation, the translocation of GLUT4, and the roles of cytokines such as TNF*α*, not forgetting how PPAR*γ*, JNK, and IKK*β* contribute to insulin resistance.

## Figures and Tables

**Table 1:** Summarised effects based on several factors involved in the mechanisms of insulin resistance and insulin signalling, including previous figures.

Factor	Effects	References
FFA	↑FFA, ↑FFA oxidation, ↑ROS, ↓glucose uptake, ↑IR	[14, 15, 18]

TNF-*α*	↑TNF-*α*, ↓tyr phosphorylation of IRS1, ↓glucose uptake, ↑IR	[22]

PPAR*γ*	↑PPAR*γ*, ↓FFA, ↑glucose uptake, ↓IR	[30]

JNK and IKK*β*	↑TNF-*α*, ↑JNK, ↑IKK*β*, ↑ser phosphorylation of IRS1, ↓tyr phosphorylation of IRS1, ↓glucose uptake, ↑IR	[12, 21]

FFA: free fatty acid; ROS: reactive oxygen species; IR: insulin resistance; TNF-*α*: tumour necrosis factor *α*; tyr: tyrosine; IRS1: insulin receptor substrate 1; PPAR*γ*: peroxisome proliferator-activator receptor *γ*; JNK: c-Jun NH_2_-terminal kinase; IKK*β*: inhibitor of nuclear factor *κ*B kinase *β*; ser: serine.
